# Access and Engagement: Distinct Roles of Digital Technology in Reducing Loneliness in Later Life

**DOI:** 10.1093/geroni/igaf122.3521

**Published:** 2025-12-31

**Authors:** Jaesung Lee, Liwei Zhang, Hee Yun Lee

**Affiliations:** University of Georgia, Athens, Georgia, United States; University of Georgia, Athens, Georgia, United States; University of Georgia, Athens, Georgia, United States

## Abstract

The growing integration of digital technologies in later life raises important issues about how both access and use influence loneliness. Although prior studies have examined technology access and technology use separately, little is known about how they jointly influence loneliness. This study addresses this gap by examining their independent and combined relationship on loneliness among older adults. Using data from 4,285 participants aged 50 and older in the 2020 Health and Retirement Study, weighted multivariate regression models were estimated. In models including each predictor separately, both device ownership (β = -0.153, p < 0.001) and digital activities (β = -0.195, p < 0.001) were associated with lower loneliness. When both predictors were included together, device ownership (β = -0.079, p = 0.004) and digital activities (β = -0.153, p < 0.001) remained significant, with activities showing the stronger association. While device ownership is linked to lower loneliness, the results from the joint model indicate that active engagement through diverse digital activities demonstrates a stronger association. This study supports the view that while ownership provides the foundation for digital inclusion, it is active and diverse engagement with these devices that more strongly enhances well-being in later life.

